# EXPLORING EARLY LIFE EXPOSURES AND THEIR IMPACT ON PHYSICAL ACTIVITY AMONG NATIVE HAWAIIAN ELDERS

**DOI:** 10.1093/geroni/igae098.4181

**Published:** 2024-12-31

**Authors:** Phoebe Hwang, Samantha Keaulana-Scott, Ilima Ho-Lastimosa, Jane Chung-Do, Kathryn Braun

**Affiliations:** University of Hawaii at Manoa, Honolulu, Hawaii, United States; University of Hawaii, Honolulu, Hawaii, United States; University of Hawaii, Honolulu, Hawaii, United States; University of Hawaii, Honolulu, Hawaii, United States; University of Hawaii, Honolulu, Hawaii, United States

## Abstract

In the United States, Native Hawaiians (NH) tend to face more barriers to meeting physical activity (PA) recommendations than other ethnic groups. Previous studies have shown that national evidence-based PA interventions do not resonate with Native Hawaiians and are not effective in increasing physical activity levels. Adverse childhood experiences (ACEs) and early life exposures significantly impact PA behaviors and health outcomes in adulthood, particularly among NH populations. The purpose of this study is to explore how early life exposures experienced by NH may affect their perceptions and behaviors towards PA. In-depth interviews were conducted with 20 NH community-dwelling elders ages 55 years and older recruited from an urban area and a rural area in O‘ahu, Hawai‘i. Interviews were audio-recorded, transcribed, and independently coded by two researchers. Three major themes were identified in this study. First, most elders were engaged in PA post-retirement, but PA type varied by the residential type (urban versus rural). Second, barriers to childhood PA were contextualized by the residential type that participants grew up in. Third, PA perspectives and behaviors were influenced by gender roles and expectations. Separately, stories of racial discrimination and ACEs naturally arose during the interviews among NH participants who were raised in urban areas. Findings suggest that PA behaviors are shaped by early life social norms, which are influenced by the residential type. Future studies should focus on exploring the connection between early life perceived discrimination and later life health behaviors.

